# Natal dispersal of Whooping Cranes in the reintroduced Eastern Migratory Population

**DOI:** 10.1002/ece3.8007

**Published:** 2021-08-11

**Authors:** Hillary L. Thompson, Andrew J. Caven, Matthew A. Hayes, Anne E. Lacy

**Affiliations:** ^1^ International Crane Foundation Baraboo Wisconsin USA; ^2^ Platte River Whooping Crane Maintenance Trust Wood River Nebraska USA; ^3^ University of Illinois Springfield Springfield Illinois USA

**Keywords:** *Grus americana*, natal dispersal, reintroduction, release location, Whooping Crane, Wisconsin

## Abstract

Natal dispersal is a key demographic process for evaluating the population rate of change, especially for long‐lived, highly mobile species. This process is largely unknown for reintroduced populations of endangered avian species. We evaluated natal dispersal distances (NDD) for male and female Whooping Cranes (*Grus americana*) introduced into two locations in central Wisconsin (Necedah National Wildlife Refuge, or NNWR, and the Eastern Rectangle, or ER) using a series of demographic, spatial, and life history‐related covariates. Data were analyzed using gamma regression models with a log‐link function and compared using Akaike information criterion corrected for small sample sizes (AIC_c_). Whooping Cranes released in the ER dispersed 261% further than those released into NNWR, dispersal distance increased 4% for each additional nesting pair, decreased about 24% for males as compared to females, increased by 21% for inexperienced pairs, and decreased by 3% for each additional year of age. Natal philopatry, habitat availability or suitability, and competition for breeding territories may be influencing observed patterns of NDD. Whooping Cranes released in the ER may exhibit longer NDD due to fragmented habitat or conspecific attraction to established breeding pairs at NNWR. Additionally, sex‐biased dispersal may be increasing in this population as there are more individuals from different natal sites forming breeding pairs. As the population grows and continues to disperse, the drivers of NDD patterns may change based on individual or population behavior.

## Graphical abstract

We evaluated natal dispersal distances for male and female Whooping Cranes (*Grus americana*) introduced into two locations in Wisconsin using a series of demographic, spatial, and life history‐related covariates. Whooping Cranes released in eastern Wisconsin dispersed 261% further than those released into central Wisconsin, dispersal distance increased 4% for each additional nesting pair, decreased about 24% for males as compared to females, increased by 21% for inexperienced pairs, and decreased by 3% for each additional year of age. Natal philopatry, habitat availability or suitability, and competition for breeding territories may be influencing observed patterns of natal dispersal distances.

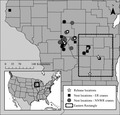

## 1 INTRODUCTION

Reintroductions of individuals into currently uninhabited areas offer unique opportunities to learn about important demographic and social processes as populations become established, grow, and expand. It is especially important to understand how individuals distribute themselves on the landscape early in a reintroduction effort to project future population range expansion and accordingly target habitat conservation (Clark et al., 2004; Lester et al., 2007). One important demographic measure of a population's ability to expand is natal dispersal (Howard, 1960). As young individuals tend to leave their birth area more than adults move between breeding attempts, this movement is important to reduce inbreeding between closely related individuals, promote gene flow between local demes, and reduce competition for resources and mates (Ferriere et al., 2000; Greenwood, 1980; Rockwell & Barrowclough, 1987). Dispersal patterns of individuals in the newly established populations can also be used to evaluate habitat suitability of an area and the utility of introducing individuals to bolster small, local populations (Krištín et al., 2006).

Characteristics of individuals including their sex and source population may influence dispersal patterns in a population. Greenwood (1980) hypothesized that sex‐biased dispersal can be due to inbreeding avoidance, and the direction of bias depends on the mating system of the species. In birds, dispersal is typically female‐biased (one notable exception is waterfowl) and is often associated with monogamy and resource defense by males (Greenwood, 1980). Dispersal patterns in a population can also depend on whether it is reintroduced, translocated, or naturally occurring (Butler et al., 2005; Calvete & Estrada, 2004; Margalida et al., 2013; Skjelseth et al., 2007). Some studies have found longer dispersal distances in reintroduced populations, which has been attributed to the tendency of reintroduced individuals to search for new territories in unfamiliar habitat or the lack of conspecific attraction due to a low population density (Margalida et al., 2013; Martín et al., 2008; Stamps, 2001). However, little is known about the dispersal patterns of reintroduced populations compared with their naturally occurring counterparts.

The Whooping Crane (*Grus americana*) is an endangered species with only one naturally formed remnant population which breeds in northern Canada and winters in coastal Texas, USA (Aransas‐Wood Buffalo Population, hereafter AWBP). Reintroduction efforts in a migratory population in the western United States (Gray's Lake Population) as well as the Florida Non‐migratory Population were deemed unsuccessful due to improper imprinting and high adult mortality. In 2001, the Whooping Crane Eastern Partnership (hereafter, the Partnership) began reintroducing Whooping Cranes east of the Mississippi River to establish a population that summered in Wisconsin and wintered in Florida, USA. This prospective population was named the Eastern Migratory Population (EMP). Prior to reintroduction efforts, no Whooping Cranes remained in this part of their range although historic records occurred (Allen, 1952; Austin et al., 2019). Additionally, in 2011 state and federal agencies began reintroducing Whooping Cranes to establish another population of Whooping Cranes in Louisiana, known as the Louisiana Non‐migratory Population (LNMP). It is important to understand dispersal patterns and population range expansion for ongoing reintroductions of Whooping Cranes to direct habitat conservation efforts and inform release strategies for captive‐reared cranes.

We report natal dispersal distances (NDD) of Whooping Cranes in the EMP and compare them with those reported for other populations of Whooping Cranes and other crane species. We also explore the potential relationship between NDD for Whooping Cranes in the reintroduced EMP and a variety of demographic (age, sex), spatial (number of nesting pairs, release area), and life history (rearing method, release method) variables that might help explain the observed pattern of natal dispersal. If cranes first establish a territory then wait to find a mate, we expect shorter NDD for cranes that start breeding at an older age, compared with younger cranes that have paired and disperse further with their mate to breed. Based on a small population size (100 individuals as of May 2019) in the EMP and a lack of sex‐biased dispersal in a similarly small AWBP (185 individuals as of 2002, during the time of Johns et al., 2005 study), we expected cranes in the EMP to also show no sex‐biased dispersal (Whooping Crane Eastern Partnership, 2019). Small naturally occurring or reintroduced populations may exhibit a lack of sex‐biased dispersal if individuals are coming from a single breeding area and one individual of each sex disperses to a breeding site and therefore have equivalent NDD. As the EMP’s breeding density has increased over time, thereby also increasing intraspecific competition for territories, we expected NDD to increase with the number of nesting pairs in the area. Lastly, rearing and release methods may affect a crane's site fidelity or familiarity with an area and potentially their NDD. For example, cranes released using different methods spend varying amounts of time in the area prior to release (approximately 0–123 days), which may affect imprinting on the area, site fidelity, and NDD. We expect cranes that fly and those that spend more time in the area prior to release to have shorter NDD than cranes that spend little to no time at the release area or cannot fly prior to release.

## 2 METHODS

### 2.1 Reintroduction techniques

**FIGURE 1:**
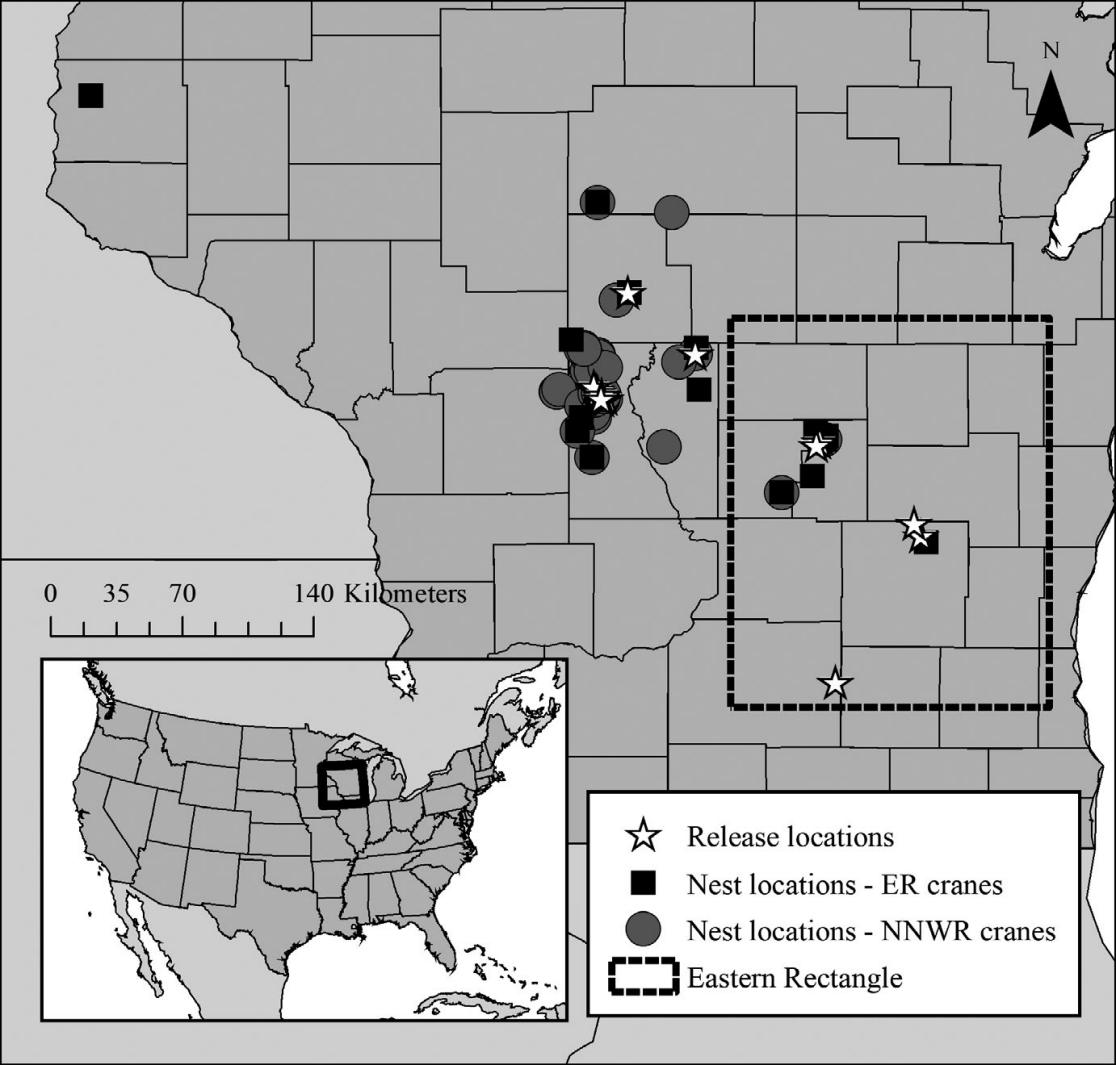
Map of release locations and nest locations of Whooping Cranes released in the Eastern Rectangle (ER) and Necedah National Wildlife Refuge (NNWR) in Wisconsin, USA. (Inset) Extent of map above

Whooping Cranes in this study hatched in the wild or were raised in captivity by either costumed caretakers (costume‐reared) or adult Whooping Cranes (parent‐reared) at the U.S. Geologic Survey's Patuxent Wildlife Research Center in Laurel, Maryland or the International Crane Foundation in Baraboo, Wisconsin. At 17–107 days of age, captive‐reared chicks were transferred to one of the two core reintroduction areas in Wisconsin, USA (Figure 1; Urbanek, Fondow, et al., 2010; Urbanek et al., 2016, M. Wellington, International Crane Foundation, pers. comm.). From 2001 to 2010, all chicks were raised at Necedah National Wildlife Refuge (NNWR) in central Wisconsin and were either taught to migrate south behind an ultralight plane (ultralight‐led) and released on the wintering grounds, or were soft‐released during fall prior to migration with other adult cranes, known as direct autumn release (hereafter DAR, Figure 1, Urbanek et al., 2014). In 2011, researchers began raising juveniles in eastern Wisconsin (hereafter the Eastern Rectangle or ER) at Horicon National Wildlife Refuge (HNWR) and White River Marsh State Wildlife Area (WRM, Figure 1; Urbanek, Zimorski, et al., 2010; Van Schmidt et al., 2014) to attempt to increase reproductive success and minimize nest abandonments due to black flies (*Simulium* spp.), which have been problematic at NNWR (Barzen et al., 2018; Converse et al., 2013; Urbanek, Zimorski, et al., 2010). From 2011 to 2012, DAR birds were raised at NNWR until they had fledged, when they were transferred to HNWR, where they were eventually released. This method is known as the modified Direct Autumn Release program, or mDAR. After 2012, DAR cranes were raised only at HNWR or WRM and the mDAR technique was discontinued. Additionally, in 2013 the Partnership began releasing parent‐reared juveniles into breeding territories of adult cranes. Parent‐reared juveniles typically spent zero or very little time in a release pen and were released directly into adult territories (hard release). Initially, all parent‐reared juveniles were released at NNWR, then as pairs became established at locations scattered throughout the range of the EMP, juveniles were released in the ER and other areas outside of NNWR (Figure 1). As of 2019, captive‐reared Whooping Cranes continued to be released in the ER.

Both release areas, NNWR and the ER, were comprised of wetland and upland habitats; however, there were some key differences with respect to size, habitat fragmentation, and wetland characteristics. NNWR is a contiguous 17,683‐ha refuge property, with sedge (*Carex* spp.) meadow wetlands, emergent marshes, prairies, oak (*Quercus* spp.) savanna, and oak‐pine (*Pinus* spp.) forest. The ER is a large 2,021,800‐ha region which includes many separate wetland properties, including HNWR and WRM. Soils in the ER were more productive than the sandy soils of NNWR. The ER had more row crop agriculture and human development and fewer forested areas than NNWR. Unlike the sedge meadows of NNWR, wetlands in the ER tended to be dominated by cattail (*Typha* spp.) vegetation.

### 2.2 Banding information and monitoring

Prior to release, each Whooping Crane was uniquely marked with colored plastic leg bands and leg‐band‐mounted VHF radio transmitters (Advanced Telemetry Systems, Isanti, MN) which enabled long‐term monitoring of individuals in the population (Urbanek, 2018). Remote transmitters (Platform Terminal Transmitters or PTTs, Global System for Mobile Communications or GSMs, Microwave Telemetry, Columbia, MD) were also deployed on the leg bands of 2–13 individuals from each cohort (130 total deployed). Whooping Cranes were monitored throughout their lives by a combination of opportunistic resightings, remote telemetry locations, and aerial or ground surveys. Each spring, biologists conducted intensive surveys to locate nesting Whooping Cranes using VHF radio telemetry from the ground as well as from a plane and recorded the identities of the individual cranes at each nest. Biologists visited accessible nest sites after eggs had hatched, were abandoned, or the pair incubated full term (30 days) without successful hatching. During nest visits, the location of each nest was collected using handheld GPS units. In areas where nests were inaccessible, coordinates were taken from a plane during an aerial survey, using the plane's GPS. Sex of each crane was determined from blood samples taken prior to release for captive‐reared juveniles, and at banding for wild‐hatched juveniles, using genetic techniques (Duan & Fuerst, 2001; Griffiths et al., 1998).

### 2.3 Statistical analysis

We assessed the influence of multiple traits of individual Whooping Cranes in the EMP on their NDD during 2005–2019. We defined an individual's natal site as either the nest where it hatched in the case of wild‐hatched individuals or the site where it was released in the case of captive‐reared individuals. We then measured the distance from the natal site to the individual's first nesting location using the “Near” tool in ArcMap version 10.6.1 (ESRI, 2011). In our study, we also included one female–female nesting pair and one male Whooping Crane who nested with a Sandhill Crane (*Antigone canadensis*), as these individuals attempted to breed, although it was not with a Whooping Crane of the opposite sex. We then examined the influence of the age of the bird at first breeding, the rearing method (costume, parent, wild), its release method (ultralight‐led, DAR or soft, mDAR, hard release, or wild‐hatched), year, release location (NNWR or ER), and whether it was establishing a new nesting territory with an inexperienced mate or was filling a gap in a previously held territory with an experienced mate (hereafter, mate experience). We used the number of breeding pairs in a given year as a proxy for breeding density. Using Pearson's product–moment correlations, we determined whether our independent variables were correlated (Dormann et al., 2013). No two independent variables with a correlation of more than |*r* > .50| were included within the same model (Dormann et al., 2013).

We generated gamma‐generalized linear models with a log‐link function, comprised of all uncorrelated variables as well as a null model, using the “glm” function in R (R Core Team, 2019). We generated 20 a priori models to examine how suites of spatially, demographically, or life history‐related variables, or combinations thereof, impacted NDD. To compare models, we used AIC_c_ and the “model.sel” tool in the “MuMIn” package in R (Barton, 2019; Burnham & Anderson, 2002; R Core Team, 2019). We used conditional model averaging for all models with an Akaike weight of 0.10 or higher using the “model.avg” tool in the “MuMIn” package (Barton, 2019; Burnham & Anderson, 2002; Wagenmakers & Farrell, 2004). We transformed parameter estimates to percent change observed in the log‐transformed dependent variable per unit increase in the independent variable following Benoit (2011). We present median values that better represent non‐normal data, as well as estimates of the mean and standard error for comparisons with other studies. All statistical analyses were done in R 3.6.0 (R Core Team, 2019).

## 3 RESULTS

As of May 2019, 309 Whooping Cranes had been released or hatched and fledged in the wild in the EMP since 2001. At that time, the current population size was ~100 cranes with 25 breeding pairs, but the population was not yet self‐sustaining (Whooping Crane Eastern Partnership, 2017, 2019). A total of 117 (71%, 57 males, 60 females) of the 165 Whooping Cranes released or wild‐fledged in WI who had reached breeding age (3 years old or older) had attempted nesting by summer of 2019. All known nests were in Wisconsin, USA, most of which were around the NNWR area (Figure 1). On average, the age at first breeding was 4.9 ± 0.3 years old for males, and 3.7 ± 0.2 years old for female Whooping Cranes. Mean NDD for all individuals in this population was 28.7 ± 4.7 km (Table 1). Mean NDD for male Whooping Cranes was 22.9 ± 6.0 km and 34.1 ± 7.3 km for female cranes (Table 1). Due to a few long dispersers, median NDD were shorter than mean NDD (median distance for all birds = 12.4 km, males = 11.7 km, females = 13.4 km, Table 1).

**TABLE 1:** Natal dispersal distances (km) of male and female Whooping Cranes in the Eastern Migratory Population, released in the Necedah National Wildlife Refuge (NNWR) or the Eastern Rectangle (ER), who nested 2005–2019

	All birds Mean ± SE (*n*) Median (range)	Males Mean ± SE (*n*) Median (range)	Females Mean ± SE (*n*) Median (range)
All release locations	28.7 ± 4.7 (117) 12.4 (0.1–357.5)	22.9 ± 6.0 (57) 11.7 (0.1–306.5)	34.1 ± 7.3 (60) 13.4 (1.1–357.5)
NNWR	15.0 ± 1.8 (92) 11.7 (0.1–78.5)	13.1 ± 2.0 (45) 11.7 (0.1–72.6)	16.9 ± 2.8 (47) 11.7 (1.1–78.5)
ER	78.7 ± 18.3 (25) 50.4 (0.3–357.5)	59.7 ± 25.5 (12) 19.0 (0.3–306.5)	96.3 ± 26.1 (13) 91.9 (3.4–357.5)

**FIGURE 2:**
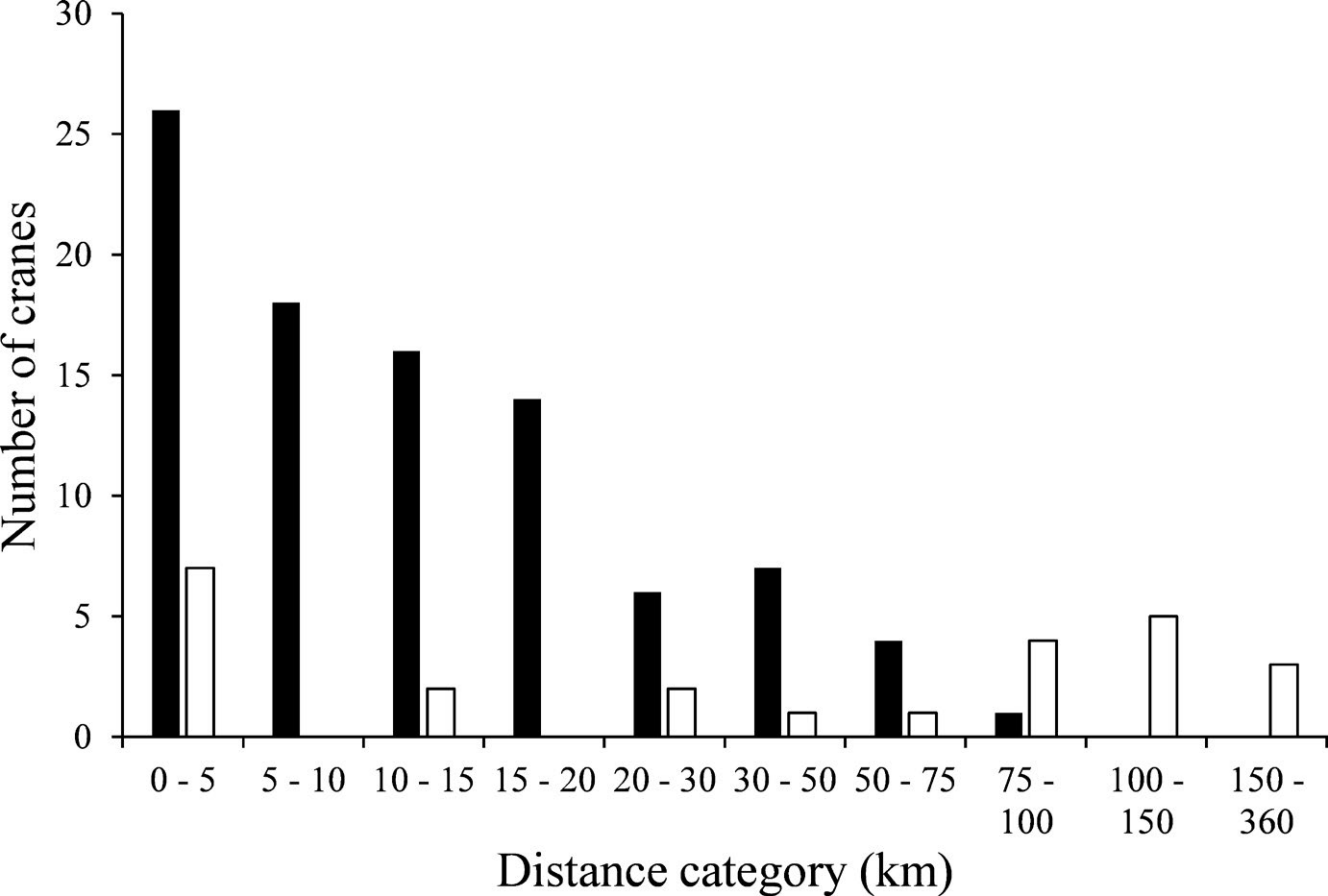
Natal dispersal distances of Whooping Cranes in the Eastern Migratory Population released in Necedah National Wildlife Refuge (black bars) (*n* = 92) and the Eastern Rectangle (white bars) (*n* = 25), 2005–2019

**FIGURE 3:**
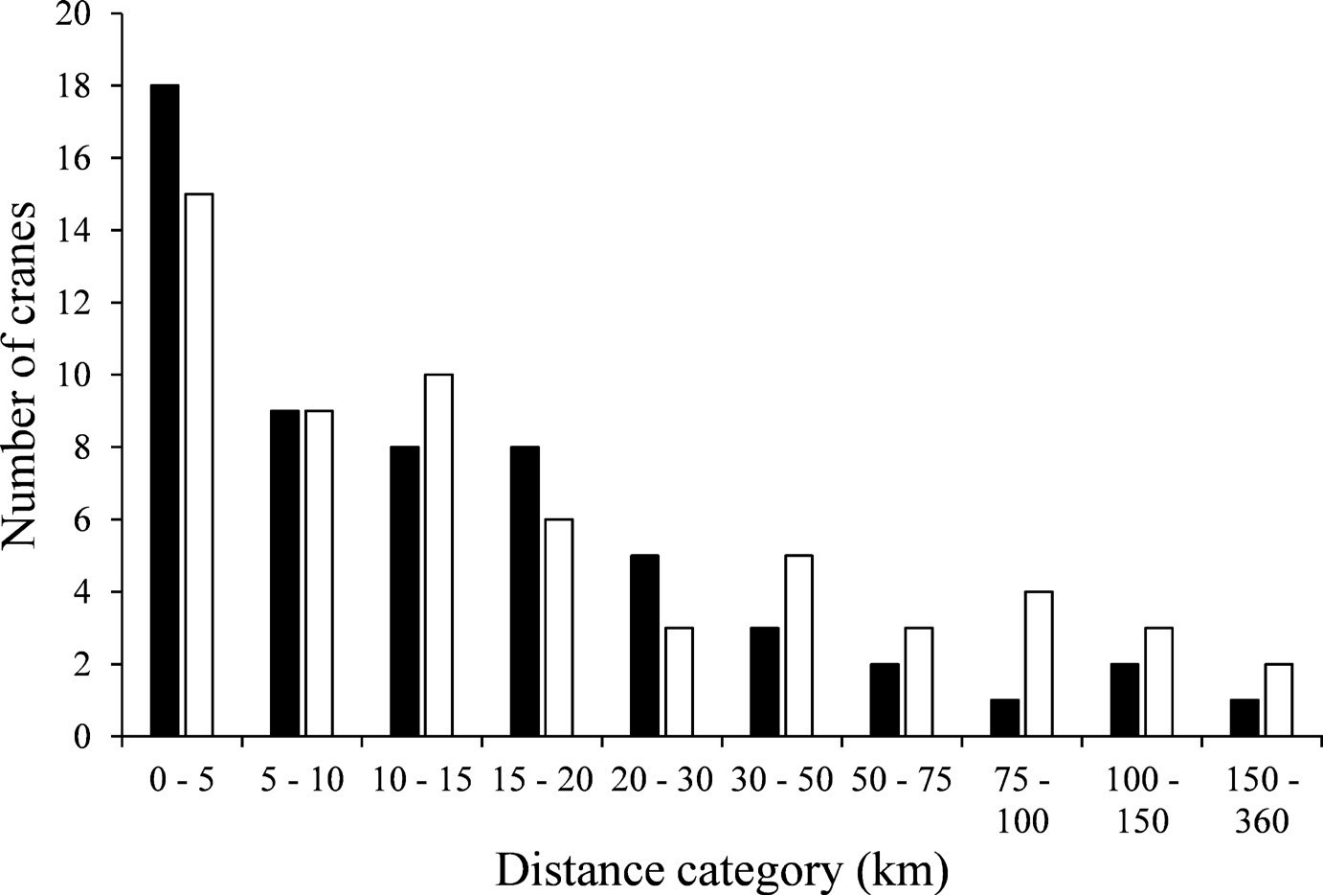
Natal dispersal distances of male (black bar) (*n* = 57) and female (white bar) (*n* = 60) Whooping Cranes in the Eastern Migratory Population, 2005–2019

The “spatial” model best predicted NDD of Whooping Cranes in the EMP and included the number of nesting pairs in the population and the individual's release location as independent variables (AIC weight = 0.254, Table 2). However, four more models, which included the spatial model with additional demographic and/or life history covariates, were within AIC_c_ delta 2 and had a model weight higher than 0.10 and therefore warrant consideration (Table 2). The second‐best model included the spatial model plus sex and had a nearly identical AIC weight to the top model (0.232, Table 2). Conditional average parameter estimates from models within AIC delta 2 suggested Whooping Cranes dispersed 4% further for each additional breeding pair in the population. Cranes released in the ER dispersed 261% further than cranes released at NNWR (Figure 2). Male Whooping Cranes first nested 24% closer to their natal site than female Whooping Cranes (Figure 3). As first breeding age increased by one year, dispersal distances shortened 3%. Lastly, individuals establishing a new territory with an inexperienced mate dispersed 21% further than those filling a gap in a previously held territory. Typically, ER cranes that nested outside of the ER tended to establish territories closer to NNWR (Figure 1). Spatial models outperformed life history and demographic models, yet some variables (sex, age at first breeding, mate experience) from life history and demographic models demonstrated value when added to spatial models. However, rearing and release methods were not included in any models with a delta weight above 0.10 predicting Whooping Crane NDD in the EMP (Table 2).

**TABLE 2:** Model selection results from generalized linear gamma regression models with log‐link functions assessing factors influencing natal dispersal distances of Whooping Cranes in the Eastern Migratory Population (2005–2019) compared using AIC_c_ (Akaike Information Criterion corrected for small sample sizes)

Model	Variables	*df*	logLik	AIC_c_	Delta	Weight
Spatial	No. Nesting Pairs +Release Location	4	−472.618	953.6	0	0.254
Spatial +Sex	No. Nesting Pairs +Release Location +Sex	5	−471.616	953.8	0.18	0.232
Spatial +Sex + Mate Experience	No. Nesting Pairs +Release Location +Sex + Mate Experience	6	−471.264	955.3	1.70	0.109
Spatial +Age	No. Nesting Pairs +Release Location +Age	5	−472.398	955.3	1.75	0.106
Spatial +Mate Experience	No. Nesting Pairs +Release Location +Mate Experience	5	−472.401	955.3	1.75	0.106
Spatial +Demographic	No. Nesting Pairs +Release Location +Sex + Age	6	−471.58	955.9	2.33	0.079
Spatial +Life History (2)	No. Nesting Pairs +Release Location +Mate Experience +Rearing Method	7	−470.977	957	3.39	0.047
Global (5)	No. Nesting Pairs +Release Location Sex +Age + Mate Experience	7	−471.208	957.4	3.85	0.037
Spatial +Release Method	No. Nesting Pairs +Release Location +Release Method	8	−471.497	960.3	6.73	0.009
Global (2)	No. Nesting Pairs +Release Location +Sex + Age +Mate Experience +Rearing Method	9	−470.344	960.4	6.78	0.009
Spatial +Life History (1)	No. Nesting Pairs +Release Location +Mate Experience +Release Method	9	−470.904	961.5	7.90	0.005
Global (4)	No. Nesting Pairs +Release Location +Sex + Release Method	9	−470.957	961.6	8.00	0.005
Global (3)	Release Location +Sex + Release Method	8	−473.397	964.1	10.54	0.001
Global (1)	No. Nesting Pairs +Release Location +Sex + Age +Mate Experience +Release Method	11	−470.258	965	11.44	0.001
Life History +Demographic + Time	Year +Sex + Age +Rearing Method	7	−483.236	981.5	27.91	0
Life History +Demographic + Time	Year +Age + Sex +Release Method	9	−481.338	982.4	28.77	0
Null Model	DV ~1	2	−503.08	1,010.3	56.67	0
Demographic	Sex +Age	4	−501.108	1,010.6	56.98	0
Life History (1)	Release Method +Mate Experience	7	−498.157	1,011.3	57.75	0
Life History (2)	Rearing Method +Mate Experience	5	−502.075	1,014.7	61.1	0

Note

Presented data include an a priori description of the model (Model), the variables included (Variables), degrees of freedom (*df*), log‐likelihood (logLik), AIC_c_ score, AIC delta, and the AIC weight.

## 4 DISCUSSION

Overall, NDD of Whooping Cranes in the EMP were comparable to those reported for other Whooping Crane populations. Cranes in the EMP dispersed slightly further (mean NDD = 28.7 ± 4.7 km, median = 12.4 km, Table 1) than cranes in the AWBP (mean NDD = 16.6 ± 1.8 km, median = 11.9 km, range = 0.3– 54.8 km, *n* = 61, Johns et al., 2005). Median dispersal distances for the EMP and AWBP were similar; however, values were more positively skewed for the EMP population as evidence of the notably higher mean. This suggests that there were more extreme high values, or lengthy dispersals, within the EMP, but otherwise the dispersal distances were very similar. These extreme values seemed to result from females released into the ER in particular (Figures 2 and 3). Whooping Cranes in the reintroduced nonmigratory population in Louisiana, USA (LNMP), dispersed further than cranes in both the EMP and AWBP (mean NDD = 47.46 ± 7.4 km, median = 47.4 km, range = 2.7–125.1 km, *n* = 33, E. K. Szyszkoski, personal communication). Longer NDD for introduced populations compared with extant populations have been found in other reintroduced populations of birds (Great Bustard, *Otis tarda*, Martín et al., 2008; Bearded Vulture, *Gypaetus barbatus*, Margalida et al., 2013). Individuals reintroduced into areas where no conspecifics currently are found may need to disperse further than extant populations to search for territories in potentially altered habitat or find mates across scattered or isolated populations.

Two models within AIC_c_ delta 2 included sex, suggesting that there is likely some sex‐biased dispersal within the EMP. From a traditional hypothesis‐testing perspective, sex was not a statistically significant predictor within the second‐best model (*p* = .169, Table 2). However, from an information‐theoretic approach, given that this variable improved the model it likely has a measurable influence on NDD (Burnham & Anderson, 1998, 2002). The lack of significance from a hypothesis‐testing perspective is due to the high variation across dispersal distances for both male (*SD* = 45.2) and female (*SD* = 56.4) Whooping Cranes. To summarize, it is likely that there is some sex‐biased dispersal within the EMP but that other variables such as the number of nesting pairs and release site account for significantly more of the variation in NDD than sex (Appendix S1).

This pattern of sex‐biased dispersal in the EMP differs from Whooping Cranes in the AWBP (Johns et al., 2005), as well as the reintroduced LNMP (mean male dispersal distance = 49.9 ± 10.9 km, mean female dispersal distance = 44.9 ± 10.2 km, E. K. Szyszkoski, personal communication), which did not exhibit sex‐biased dispersal. However, Johns et al. (2005) did not use a statistical approach to compare NDD of Whooping Cranes in the AWBP. Additionally, sex‐biased dispersal has been documented in other species of cranes with females dispersing longer distances than males (Sandhill Cranes, Nesbitt et al., 2002, Hayes, 2015; Red‐crowned Cranes, *Grus japonensis*, Masatomi, 2003).

Notably, 70 of the 117 breeding Whooping Cranes in the EMP paired with mates who were released from the same location; thus, both members of a pair dispersed the same distance from their core release locations to their nest location, resulting in no difference in NDD. Therefore, sex‐biased dispersal in the EMP could become more apparent over time as more individuals pair with mates from different natal areas. Whitfield et al. (2009) found the same pattern of NDD at the beginning of a reintroduction of released White‐tailed Eagles (*Haliaeetus albicilla*) in western Scotland. Natal dispersal distances of male White‐tailed Eagles did not change over the 25+ year study; however, female NDD increased as the population expanded (Whitfield et al., 2009). In recent years, more EMP Whooping Cranes have hatched in the wild and the Partnership released captive‐bred birds at new sites, thus expanding the distribution of birds throughout Wisconsin. As birds from different release areas form pairs, we are able to measure differences in NDD between males and females. It appears females are beginning to disperse further than males, which could lead to increasing sex‐biased dispersal patterns in the future. It is also possible that sex‐biased dispersal distances have developed along with population growth in the AWBP since Johns et al. (2005) completed fieldwork in 2002. Johns et al. (2005) hypothesized that small population size and decreased opportunity to find mates with increased dispersal distances were driving the lack of sex‐biased dispersal. Movements by juvenile birds prior to nesting, however, were not well documented. There was no evidence of depredation, competition, or habitat changes influencing patterns of sex‐biased dispersal in the AWBP (Johns et al., 2005). Alternatively, the abundance of appropriate breeding habitat in and around Wood Buffalo National Park may be sufficient to limit the need for distant dispersals, while suboptimal or fragmented habitat in the EMP may promote longer distance dispersals (Divoky & Horton, 1995).

The number of nesting pairs in the population and the age at which a crane first nested affected NDD. In years with more breeding pairs in the population, first‐time nesters dispersed further from their natal area, suggesting territories closer to release sites were occupied, and individuals had to move further to find suitable, unclaimed breeding habitat. However, if an individual waited to breed and nested for the first time at an older age, they had a shorter NDD. Nesbitt and Tacha (1997) hypothesized that Sandhill Cranes must first occupy a territory, then wait for an available mate. There may be three strategies for cranes to find a mate and a high‐quality breeding territory: (1) occupy a territory close to the natal site and wait to find a mate, potentially breeding at an older age but with a shorter NDD, (2) first find a mate, then search together for a vacant breeding territory, potentially breeding sooner but further from the release area with a longer NDD, or (3) remain near the natal site until they can out‐compete another male for an established mate and territory. Relatedly, Pasinelli and Walters (2002) found that male Red‐cockaded Woodpeckers (*Picoides borealis*) were more likely to defer breeding in favor of remaining as a helper in higher quality territories given an increased probability of eventually inheriting that breeding site. Though Whooping Cranes have a very different social system, it is possible that remaining nearer to the natal site has a cost in terms of age at first breeding but a benefit in terms of habitat quality. However, as a long‐lived species, the cost in age at first breeding may be relatively small for Whooping Cranes compared with smaller short‐lived species.

Release location had the largest effect on NDD of Whooping Cranes in the EMP. There are potentially a variety of factors contributing to long NDD of ER cranes and the formation of territories of ER cranes near NNWR. These factors may include the formation of crane pairs from different natal areas, differences in habitat availability near release locations, and the influence of conspecific association and higher population density at NNWR than in the ER. In this study, only 13 of the 117 breeding Whooping Cranes nested for the first time with a mate released in a different region (NNWR vs. ER), and pairs of ER Whooping Cranes have established territories closer to NNWR. Three of the four breeding mDAR cranes established territories outside of the ER and near NNWR and may be imprinted on areas they used prior to fledging, when they were moved into the ER. Though, not a variable in the top models, data suggested that dispersal distances for mDAR‐released birds were generally higher and statistically different from ultralight (UL) released birds (*p* = .048, ANOVA with the Tukey HSD test).

If there is more appropriate or contiguous nesting habitat in NNWR than in the ER, Whooping Cranes may be dispersing long distances to locate available habitat, regardless of their natal area. NNWR is comprised of large contiguous sedge meadow wetlands adjacent to open uplands, managed as a single property with minimal human disturbance or activity. Unlike NNWR, the ER is a much larger area including row crop agriculture, higher levels of human development, and many isolated wetlands dominated by cattails. Due to this difference in the landscape, cranes at NNWR may not have to disperse as far as cranes in the ER to find appropriate nesting habitat near their natal areas.

Attraction to conspecifics could also be contributing to the pattern of ER cranes establishing territories near NNWR. As of 2019, most Whooping Cranes in the EMP summer in or around NNWR, with a smaller contingent of birds in the ER. Prior to 2011, the Partnership released large cohorts of Whooping Cranes at NNWR to establish the original core population (5–29 birds released per year). Beginning in 2011, when the Partnership focused on releasing cranes in the ER, there were smaller cohort sizes (3–18 birds released per year) and multiple release locations within a larger area (Whooping Crane Eastern Partnership, 2017). It is possible this change contributed to a slower establishment of breeding pairs of Whooping Cranes in the ER, less conspecific attraction to the area, and resulted in longer NDD as birds initially established territories near breeding pairs at NNWR. A similar pattern was found in reintroduced Griffon Vultures (*Gyps fulvus*) in France, where birds dispersed differently among release sites, selecting areas near large established populations (Le Gouar et al., 2008).

ER cranes selecting territories near NNWR, either due to conspecific attraction or a flexibility of this population to seek out appropriate breeding habitat, may have population‐level consequences. Due to avian‐feeding black flies (*Simulium* spp.) contributing to widespread nest‐abandonment at NNWR, continued establishment of Whooping Crane territories in that area could continue to limit self‐sustainability in the EMP (Barzen et al., 2018; Converse et al., 2013; Urbanek et al., 2010). With continued releases of captive‐reared individuals in the ER, there may be a stronger influence of conspecific attraction on breeding territory establishment and shorter NDD of ER cranes in the future. Ultimately, a shift in high density breeding areas from NNWR to the ER may contribute to greater productivity in the EMP. Continuing to monitor NDD as the number of breeding pairs in the ER increases will help us better understand the influence of conspecific attraction as well as habitat on crane behavior across the two core release areas. The information gathered in this study will help inform managers of this endangered species with regard to identifying appropriate nesting habitat as well as the logistics of future releases of captive‐reared individuals into this population.

## CONFLICT OF INTEREST

None declared.

## AUTHOR CONTRIBUTIONS

**Hillary L. Thompson:** Conceptualization (equal); Data curation (lead); Formal analysis (supporting); Investigation (lead); Project administration (lead); Writing‐original draft (lead); Writing‐review & editing (equal). **Andrew J. Caven:** Formal analysis (lead); Writing‐original draft (supporting); Writing‐review & editing (equal). **Matthew A. Hayes:** Conceptualization (equal); Investigation (supporting); Writing‐original draft (supporting); Writing‐review & editing (equal). **Anne E. Lacy:** Conceptualization (equal); Funding acquisition (lead); Writing‐original draft (supporting); Writing‐review & editing (equal).

## ETHICS STATEMENT

This research was conducted in compliance with the *Ethical Guidelines for Statistical Practice*. No wild birds were handled specifically for this project.

## Supporting information

Appendix S1

## Data Availability

These data are available at https://doi.org/10.5061/dryad.9w0vt4bf0.
