# Supplementary material for: Natal dispersal of Whooping Cranes in the reintroduced Eastern Migratory Population
Source: Ecol Evol. 2021 Aug 11;11(18):12630–8. doi: 10.1002/ece3.8007 (PMC8462167; doi:10.1002/ece3.8007)
Supplement: Supplementary file 1 — Appendix S1 [file ECE3-11-12630-s001.docx]

APPENDIX 1. Top performing Generalized Linear Gamma Regression Models (AIC weight > 0.10) presented with both model-averaged (x̄ *B*) and standard (*B*) parameter estimates for independent variables in relation to log-transformed natal dispersal distances (via log-link function). Model averaged parameter estimates are derived from all models above an AIC weight of 0.10 and within an AIC delta of 2.

| Model | Coefficient | *x̄ B* | *B* | *SE* | *t* | *p* |  |
| --- | --- | --- | --- | --- | --- | --- | --- |
| Spatial | (Intercept) | --- | 1.990 | 0.277 | 7.18 | <0.001 | *** |
|  | Release Area (Rectangle) | 1.285 | 1.296 | 0.267 | 4.86 | <0.001 | *** |
|  | No. Nesting Pairs | 0.039 | 0.040 | 0.015 | 2.72 | 0.008 | ** |
|  |  |  |  |  |  |  |  |
| Spatial + Sex | (Intercept) | --- | 2.141 | 0.292 | 7.34 | <0.001 | *** |
|  | No. Nesting Pairs | 0.039 | 0.038 | 0.015 | 2.63 | 0.010 | ** |
|  | Sex (Male) | -0.272 | -0.266 | 0.192 | -1.38 | 0.170 |  |
|  | Release Area (Rectangle) | 1.285 | 1.289 | 0.266 | 4.85 | <0.001 | *** |
|  |  |  |  |  |  |  |  |
| Spatial + Sex + Mate Experience | (Intercept) | --- | 1.984 | 0.401 | 4.95 | <0.001 | *** |
|  | No. Nesting Pairs | 0.039 | 0.038 | 0.015 | 2.49 | 0.014 | * |
|  | Mate Experience (Inexperienced) | 0.186 | 0.209 | 0.262 | 0.80 | 0.427 |  |
|  | Sex (Male) | -0.272 | -0.285 | 0.195 | -1.46 | 0.146 |  |
|  | Release Area (Rectangle) | 1.285 | 1.286 | 0.273 | 4.70 | <0.001 | *** |
|  |  |  |  |  |  |  |  |
| Spatial + Age | (Intercept) | --- | 2.089 | 0.336 | 6.22 | <0.001 | *** |
|  | No. Nesting Pairs | 0.039 | 0.043 | 0.015 | 2.80 | 0.006 | ** |
|  | Age | -0.034 | -0.034 | 0.059 | -0.58 | 0.565 |  |
|  | Release Area (Rectangle) | 1.285 | 1.239 | 0.282 | 4.39 | <0.001 | *** |
|  |  |  |  |  |  |  |  |
| Spatial + Mate Experience | (Intercept) | --- | 1.860 | 0.396 | 4.70 | <0.001 | *** |
|  | No. Nesting Pairs | 0.039 | 0.039 | 0.015 | 2.58 | 0.011 | * |
|  | Mate Experience (Inexperienced) | 0.186 | 0.163 | 0.264 | 0.62 | 0.538 |  |
|  | Release Area (Rectangle) | 1.285 | 1.294 | 0.276 | 4.70 | <0.001 | *** |

s
